# Lead(II)-Azido Metal–Organic Coordination Polymers: Synthesis, Structure and Application in PbO Nanomaterials Preparation

**DOI:** 10.3390/nano12132257

**Published:** 2022-06-30

**Authors:** Jaber Dadashi, Mohammad Khaleghian, Younes Hanifehpour, Babak Mirtamizdoust, Sang Woo Joo

**Affiliations:** 1 Department of Chemistry, Faculty of Science, University of Qom, Qom P.O. Box 37185-359, Iran; jaber_dadashi_95@yahoo.com (J.D.); mohammad.khaleghian95@gmail.com (M.K.); babakm.tamizdoust@gmail.com (B.M.); 2 Department of Chemistry, Sayyed Jamaleddin Asadabadi University, Asadabad 6541861841, Iran; 3 School of Mechanical engineering, Yeungnam University, Gyeongsan 712-749, Korea

**Keywords:** coordination polymer, lead(II) complex, azide, ultrasonic irradiation, lead oxide, sonochemical synthesis

## Abstract

The current study aims to explain recent developments in the synthesis of Pb(II)-azido metal-organic coordination polymers. Coordination polymers are defined as hybrid materials encompassing metal-ion-based, organic linkers, vertices, and ligands, serving to link the vertices to 1D, 2D, or 3D periodic configurations. The coordination polymers have many applications and potential properties in many research fields, primarily dependent on particular host–guest interactions. Metal coordination polymers (CPs) and complexes have fascinating structural topologies. Therefore, they have found numerous applications in different areas over the past two decades. Azido-bridged complexes are inorganic coordination ligands with higher fascination that have been the subject of intense research because of their coordination adaptability and magnetic diversity. Several sonochemical methods have been developed to synthesize nanostructures. Researchers have recently been interested in using ultrasound in organic chemistry synthetics, since ultrasonic waves in liquids accelerate chemical reactions in heterogeneous and homogeneous systems. The sonochemical synthesis of lead–azide coordination compounds resulted from very fantastic morphologies, and some of these compounds are used as precursors for preparing nano lead oxide. The ultrasonic sonochemistry approach has been extensively applied in different research fields, such as medical imaging, biological cell disruption, thermoplastic welding, food processing, and waste treatment. CPs serve as appropriate precursors for preparing favorable materials at the nanoscale. Using these polymers as precursors is beneficial for preparing inorganic nanomaterials such as metal oxides.

## 1. Introduction

### 1.1. Coordination Polymer

**Figure 1:**
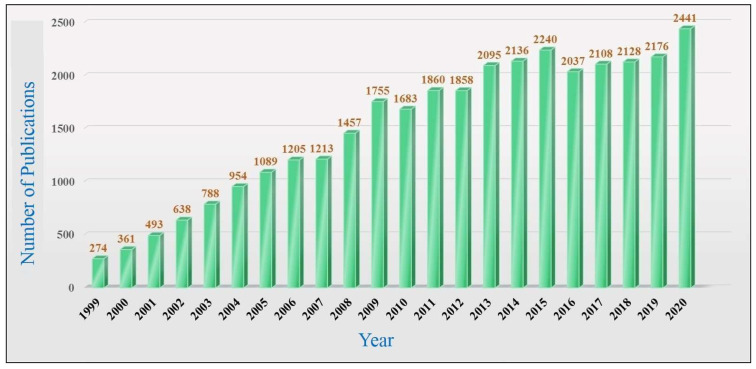
Progress in interest in synthesizing coordination polymers (CPs) (Source: Scopus).

**Scheme 1:**
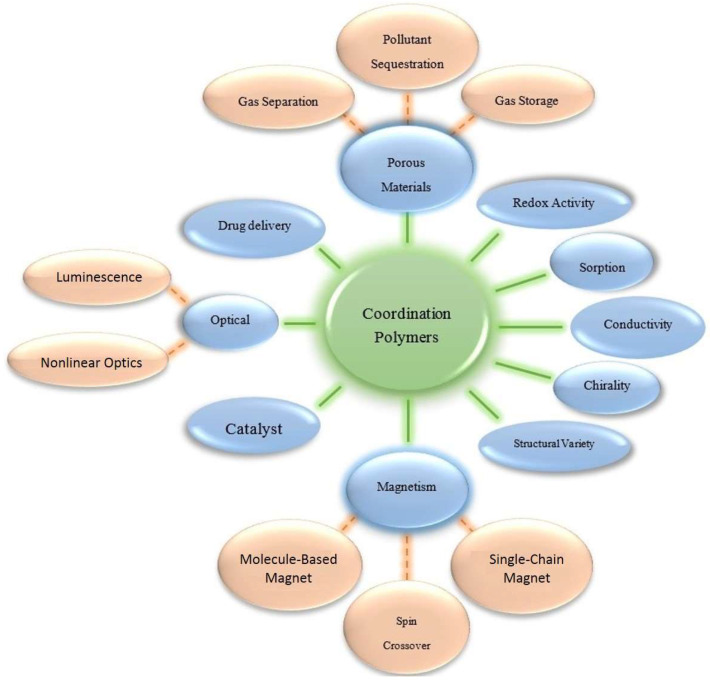
Some of the applications and potential properties of coordination polymers.

Metal coordination polymers (CPs) and complexes have fascinating structural topologies. Therefore, they have found numerous applications in different areas over the past two decades (Figure 1). CPs are defined as hybrid materials encompassing metal-ion-based, organic linkers, vertices, and ligands, linking the vertices to 1D, 2D, or 3D periodic configurations [1]. Researchers from both the material science and chemistry fields have recently been interested in these polymers. Through the appropriate selection of organic linker building blocks and metal ions, CP properties and structures can be well-organized. A broad scope of properties has been observed in these materials that ranges from luminescence [2,3,4,5] to magnetism [6], nonlinear optics [7], and electrical conductivity [8] (Scheme 1). Researchers have shown great attention to polymers having porous structures, named porous coordination polymers (PCPs) or metal–organic frameworks (MOFs). They can obtain different architectures [9,10] because of their inherent porosity, sizeable inner surface area, and the ability to tune pore topologies and sizes [11,12,13,14,15], converting these materials into potentially suitable materials for gas storage and adsorption [16,17,18,19], drug delivery [20,21,22], liquid and gas separation [23,24,25,26,27,28,29,30,31,32,33,34,35,36,37,38,39,40,41], sensor technology [42,43] heterogeneous catalysis [44,45,46,47,48,49,50,51,52,53], hosting nanoparticles or metal colloids [54] or polymerization reactions [55], water sorption for heat transformation [56,57,58,59,60,61,62], photoreactivity and photosalient effects, and pollutant sequestration [63]. These potential applications are primarily dependent on particular host–guest interactions.

### 1.2. Lead

#### 1.2.1. Lead Coordination Chemistry

Most lead coordination compounds have been reported as oxidation (II). Meanwhile, a small number of compounds containing Pb(IV) have been determined so far. Because of its relatively large ion (radius 1.23 Å to 1.43 Å), Pb(II) has a wide variety of coordination numbers in its complexes. Accordingly, Pb(II) complexes represent coordination numbers from 1 to 12, and O, N, S, P, Br, I, and Cl atoms are ordinary donor atoms in their coordination complex. In many cases, one of the complexities of lead coordination chemistry is the considerable distance between lead atoms and ligand donor atoms, making it hard to determine the metal’s precise coordination number. Despite the suitability of the Van der Waals radii sum to measure the upper limit of the distance related to the metal–ligand secondary interaction, higher values of the radius for Pb(II) must be considered.

#### 1.2.2. Pb(II) Electron Pair 6s^2^ in Coordination Structures

**Scheme 2:**
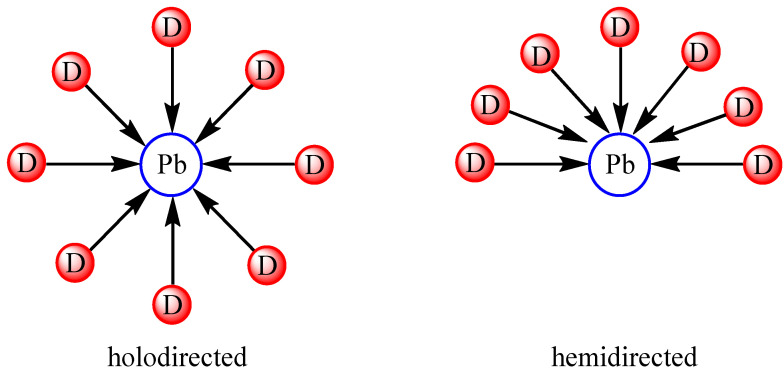
The stereochemical effect of the 6s pair on the coordination sphere (a ligand’s D donor atom).

The role of unbonded electron pairs of lead(II) in the coordination geometry was investigated by crystallographic research works and optimizations of molecular orbital calculations. The structural effects of pairs of single electrons on intermediate heavy metals such as Bi(III), Ti(I), and Pb(II) have been recently frequently studied [64]. Some contemporary researchers have studied the effect of single-electron pairs on lead(II) and have examined their structural effects. Lead(II) has the electronic structure [Xe]4f^14^5d^10^6s^2^. Due to relativistic effects, which are maximum at Au(I) and operative in other close 6p metals such as lead, the 6s orbital is contracted and stabilized. This stabilized 6s pair reduces its participation in the chemistry of the element (becoming an “inert-pair”), and this explains why inorganic Pb forms compounds in a lower oxidation state (less by two) than would be expected from its group number [65]. The apparent reticence of the 6s electrons to play a role in the chemistry of the element may also affect the stereochemistry of Pb(II) complexes. This influence can be understood in simple hybridization or valence-shell electron-pair repulsion arguments. By using the former approach, it seems that the 6s orbital, despite its stabilization, can hybridize with the 6p orbitals to give a “stereochemically active” 6s electron pair (or stereochemically active lone electron pair, *SALEP*) occupying one position in the coordination sphere of the metal. Because the pair is not directly detectable, its presence is normally identified by a void in the distribution of the coordination bonds (*hemidirected* coordination, see Scheme 2). If hybridization does not occur and the pair has only an s character, then it is “stereochemically inactive”, and the complex does not show a gap or void in the bond distribution (*holodirected* coordination, see Scheme 2) [65,66].

#### 1.2.3. Holodirected and Hemidirected Structures

Studies have reported two Pb(II) compound structures: holodirected and hemidirected. In holodirected structures, the ligand atoms bond is scattered throughout the Pb(II) coordination space. However, in the hemidirected structure, the ligand atoms bond is diffused only in one part of the Pb(II) coordination space (Scheme 2) [67,68]. In these compounds, space is found in the distribution of ligands around the cation. Shimoni Livni et al. reported that all Pb(IV) compounds have a holodirected structure [65]. However, holodirectional and hemidirectional geometric structures are observed in the case of divalent lead despite a nonbonded electron pair. All lead Pb(II) compounds by coordination numbers 2 to 5 have a hemidirected geometric structure, while the coordination numbers 9 and 10 of lead have a holodirected geometric structure. Both of the structures have been reported for coordination numbers six to eight. However, the holodirected structure is more suitable for coordination numbers six to eight when bulky and soft ligands are present. Thus, with the high number of coordinated ligands near Pb(II), the valence layer’s electron-pair activity effects diminish [69]. Several factors affect the activity or inactivity of the lead ion layer electron pair, such as the coordination number, type of ligands in terms of hardness and softness, attraction or repulsion between ligands, electrical charge, and the size of ligands [70]. Overall, two rules can be extracted using these factors: First, small coordination numbers, hard ligands (N or O donors), and gravitational interactions among ligands or multidentate ligands are all suitable for hemidirected structures. Second, large coordination numbers, soft ligands (Br-, Cl-, and I-), and repulsive interactions among ligands result in holodirected structures. Although these are not error-free rules, they could be beneficial in designing Pb(II) complexes or certain structural features [71]. The amount of energy required to convert a hemidirected structure to a pressurized holodirected one without strong interactions between ligands is calculated as 8–12 kcal/mol.

### 1.3. Azide

**Scheme 3:**
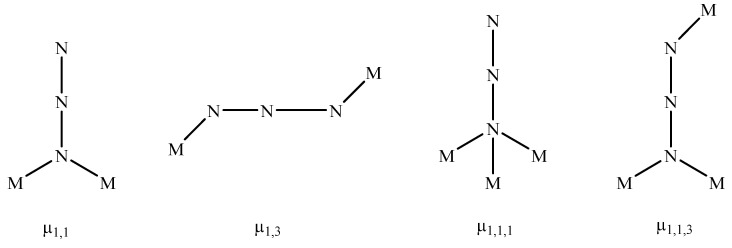
Usual bridging states of azido ligand.

Bridge-building ligands are the main factor for preparing CPs and multicore complexes. There are various bridging ligands such as OH^−^, S_2_^−^, SH^−^, NH_2_, NH^−^, N_3_^−^, halides, and pseudohalides (N_3_, NCO, NCS, and NCSe). In coordination chemistry, halide-like ligands are significant and valuable agents used as bridge-building ligands. Like other bridge-making ligands, they offer multinucleated complexes with highly diversified structures and fascinating magnetic characteristics. Schiff base ligands containing N and O donors are extensively employed for building multinucleated complexes with fascinating configurations. Azido-bridged complexes are inorganic coordination ligands with higher fascination that have been the subject of intense research because of their coordination adaptability and magnetic diversity. In these structures, the azide anion can present a behavior like a tridentate linking ligand (μ_1,1,1_-N_3_ or μ_1,1_,_3_-N_3_) and a bidentate linking ligand (end-to-on, μ_1,1_-N_3_ “EO”, or end-to-end, μ_1,3_-N_3_, “EE”, linking states) (Scheme 3) [72]. Bidentate coordination can mediate antiferromagnetic and ferromagnetic interactions among the metallic ions. Furthermore, inorganic azides serve as a significant category of solid compounds. These modes might be present concurrently in the same complex in various alternating sequences. In addition to the availability of coligands, the properties mentioned above have resulted in different 1D and 2D arrangements with fascinating magnetic properties or polymeric topologies. Some of these topologies have practical significance as explosives, some are important as industrial chemical materials, and some could be used as valuable photographic materials at low temperatures. Lead azide is a principal explosive substance with high sensitivity in primer, fuses, and blasting caps. Alkali metal azide systems are benign in behavior. It is important to understand the basic characteristics and electron configuration of alkali metal azides in order to understand their behavior. Metal–azo systems with a second linking ligand represent an innovative approach toward a high-dimensional topology. This strategy provides a growing number of 2D and 3D systems. The second widely used bridging ligands include pyridyl-based diatopic ligands such as 4,4′-bipy, pyrazine, and their equivalents [73].

## 2. Sonochemical Method

Using sonochemistry, chemical reactions can be studied under an ultrasound frequency radiation influence (10–20 MHz). Compared to conventional energy sources (e.g., light, heat, and electric potential), relatively uncommon reaction settings are provided by ultrasonic sonochemistry. This approach provides a short period of very high pressures and temperatures in liquids, which would not be provided by other approaches [74]. Several sonochemical methods have been developed to synthesize nanostructures; among these methods, we can mention sonochemical reduction, ultrasound-induced deposition, sonoelectrochemistry, ultrasonic spray p3yrolysis, etc. Almost all sonochemical techniques are based on the physical effects of ultrasound. The principle that causes the modification of nanostructures in the sonochemical process is acoustic cavitation. When the particle dimensions are much smaller than the applied ultrasonic wavelength, no direct interaction occurs between them. Therefore, high-energy ultrasound needs to be applied, which leads to the appearance of a cycle of continuous low pressure (rarefaction) and high pressure (compression). In the rarefaction, the pressure collapses under the vapor pressure of the solvents, and prepared holes grow the bubbles to more than tens of micrometers. The bubbles become unstable and break down when they achieve their most extreme size. This continuous procedure of bubble preparation, growth, and collapse breakdown is called cavitation, produces “hot spots,” and it prompts the quick release and gains of energy with cooling, up to a temperature of ~5000 K and heating rate of >1000 Ks^−1^, and pressure of ~1000 bar. At a further distance of 200 nm, profound shear forces occur. So many parameters such as temperature, viscosity, vapor pressure, acoustic intensity and frequency, chemical reactivity, and the gas atmosphere affect the preparation of the cavities, and just a tiny amount of energy is transferred into the holes [75]. These extreme situations could drive chemical reactions. However, they can cause nanosized structure formation, generally by the instant development of crystallization nuclei. Moreover, it has been extensively employed for fabricating various complexes with nanosized constructions [76]. Researchers have recently been interested in using ultrasound in organic chemistry synthetics since ultrasonic waves in liquids lead to the acceleration of chemical reactions in heterogeneous and homogeneous systems [77]. Usually, for sonochemistry reactions, volatile organic solvents are usually not suitable because the collapse intensity of cavities decreases in high vapor pressure. These methods can produce different nanostructures and nanoparticles of different sizes and shapes by controlling the synthesis processes conditions and components. These methods are used to synthesize various nanoparticles, including metal nanoparticles, metal oxide nanostructures, quantum dots, porous nanostructures, hollow nanostructures, etc. In addition to nanoparticle synthesis, sonochemical methods have been widely used in the deposition and accumulation of nanoparticles on surfaces and even on other nanoparticles, indicating the potential of these nanotechnology methods. The ultrasonic sonochemistry approach has been extensively applied in different research fields, such as medical imaging, biological cell disruption, thermoplastic welding, food processing, and waste treatment. Some studies have focused on ultrasonic sonochemistry applications to the chemistry of solid materials. Many supramolecular polymer–metal compounds and organic reactions have been conducted under ultrasonic sonochemistry irradiation during a short reaction time with large yields [78]. The aspects of room temperature preparation, energy-efficiency, fast formation, and being environmentally friendly are the initial aim of utilizing a sonochemical procedure in MOF science. Furthermore, crystalline nanoparticles that are regularly obtained by the sonochemical approach possess significant advantages and applications.

**Scheme 4:**
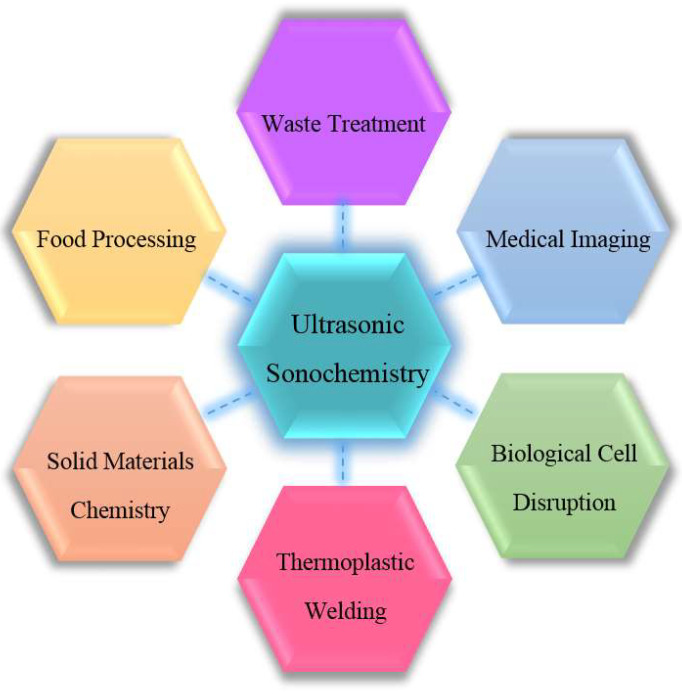
Application of ultrasonic sonochemistry method in different research fields.

Several methods can be employed to synthesize lead–azide nanoparticles, such as sonochemistry, hydrothermal, solvothermal, etc. Researchers’ results reveal that ultrasonic synthesis can be employed successfully as a simple, efficient, low-cost, environmentally friendly, and very promising method for the fabrication of lead–azide coordination polymers with tunable size and morphology by varying the reaction conditions [79,80]. Moreover, the results show that particle size decreases with increasing ultrasound power. Interestingly, the sizes and morphologies of the nanostructures depend on the concentration of the initial reagents and the power of the ultrasound irradiation used [81,82]. Scheme 4 provides application of ultrasonic sonochemistry method in different research fields.

## 3. Synthesis of Lead Azide Coordination Polymers

**Figure 2:**
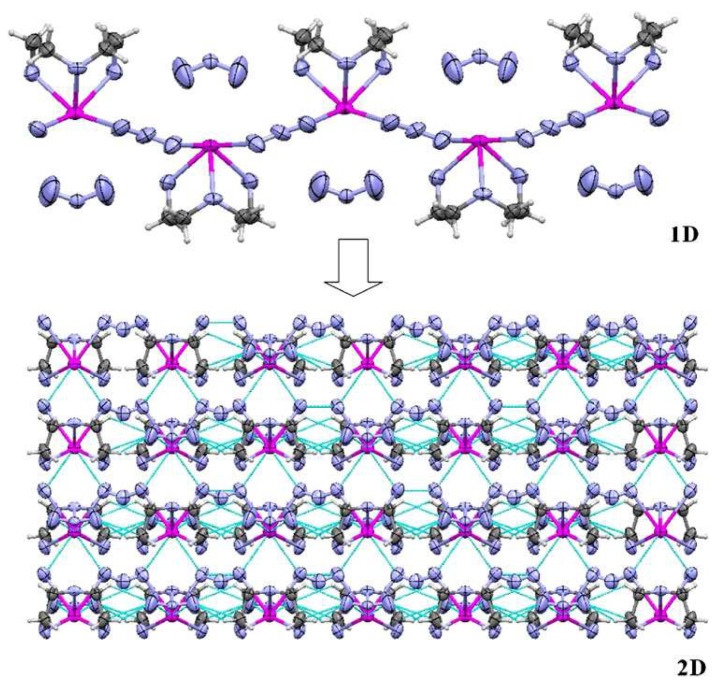
From 1D CP in [Pb(baea)(N_3_).(N_3_)]_n_ to 2D CP with supramolecular interactions. Reprinted with permission from [83]. Copyright 2012, Springer Nature.

Fun et al. (2012) synthesized sonochemically a nanostructure of a novel polymeric Pb(II) compound possessing azide anions [Pb(baea)(N_3_).(N_3_)]_n_ (baea: bis(2-aminoethyl amine) [83]. Single-crystal XRD demonstrated that the coordination number of lead(II) ions was five (PbN_5_), and it held a hemidirected coordination structure. Each lead atom was chelated by three nitrogen atoms of the “baea” ligand with Pb-N distances of 2.457(6), 2.465(6), 2.465(6) Å, and two nitrogen atoms of azide anions with a Pb-N distance of 2.905(8), 2.905(8) Å. The coordination modes of the azide anions were µ_1,1,3,3_. Furthermore, they showed that the 1D CPs interacted in supramolecular way and had weak Pb---N interactions for creating 2D supramolecular structures (Figure 2).

**Figure 3:**
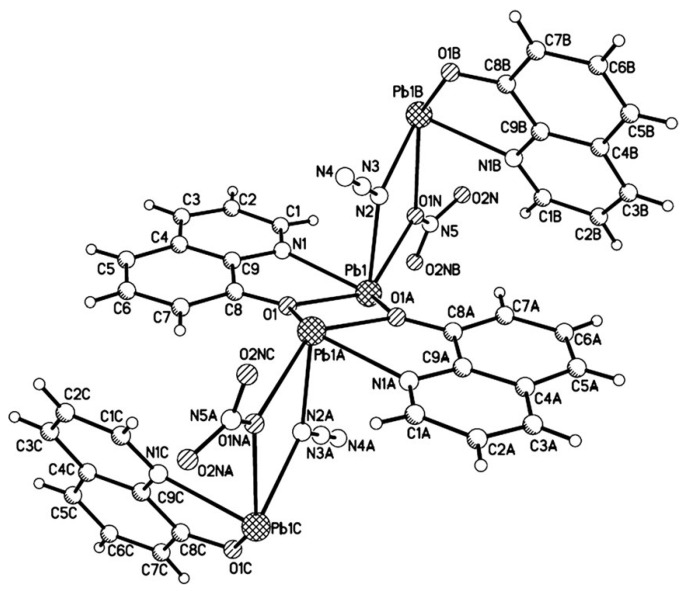
ORTEP diagram and representation of Pb(II) space in [Pb_2_(µ-N_3_)(µ-NO_3_)L_2_]_n_. Reprinted with permission from [79]. Copyright 2013, Elsevier Ltd., Amsterdam, The Netherlands.

Soltanian Fard et al. (2013) developed a novel 2D double-chain Pb(II) CP [Pb_2_(µ-N_3_)(µ-NO_3_)L_2_]_n_ sonochemically [79]. The polymer’s coordination number was 5, and Pb atoms had coordination with one N atom of the ligand, one O atom of the NO_3_^−^ anion, two O atoms of the ligand’s OH group, and one N atom of the azide anion (Figure 3). Calcination of the CP under an air atmosphere at 400 °C resulted in PbO nanoparticles identified by XRD and SEM.

**Scheme 5:**
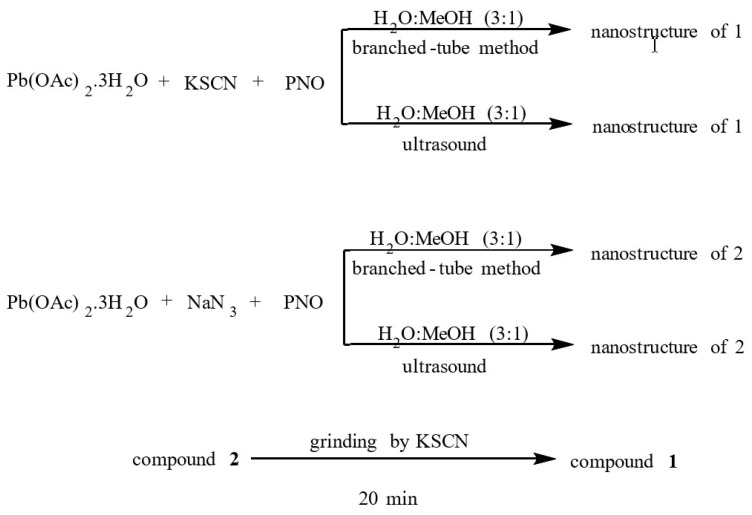
Schematic graphs of synthetic methods.

Morsali and Aboutorabi (2016) reported a simple protocol for the preparation of two novel Pb(II) mixed-ligand coordination compounds [Pb(PNO)(SCN)]_n_ and [Pb(PNO)(N_3_)]_n_ (PNO: picolinic acid N-oxide) using the sonochemical technique (Scheme 5) [82]. As shown by evaluating the structural transformation of [Pb(PNO)(SCN)]_n_, [Pb(PNO)(N_3_)]_n_ and converting [Pb(PNO)(N_3_)]_n_ to [Pb(PNO)(SCN)]_n_, there was a phase conversion from monoclinic P2_1_/n to orthorhombic P2_1_2_1_2. This conversion can be considered as an irretrievable transformation from 3D to 2D. This manner’s remarkable advantages are the need for shorter reaction times and the CP production by better nanosized outcomes.

**Figure 4:**
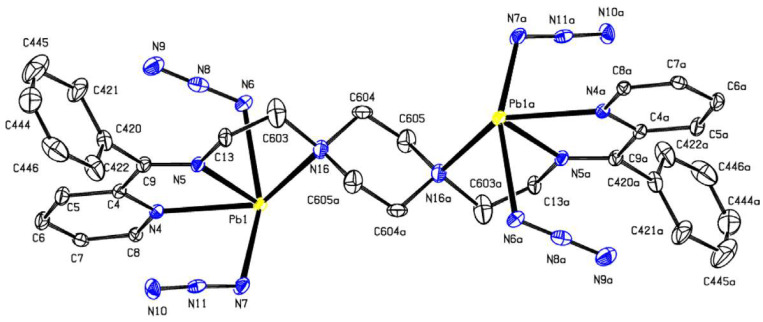
ORTEP view of [Pb_2_(pbap)(N_3_)_4_] (1) with atom-numbering scheme and 20% probability ellipsoids for all non-H atoms. Reprinted with permission from [84]. Copyright 2010, Elsevier Ltd., Amsterdam, The Netherlands.

A simple procedure was proposed by Chattopadhyay et al. (2010) for the synthesis of two pentacoordinated dinuclear Pb(II) complexes, namely [Pb_2_(pbap)(NCS)_4_] and [Pb_2_(pbap)(N_3_)_4_] (pbap: N-((1-pyridine-2-yl)benzylidene)-N-[2-(4-{2-[((1-pyridine-2-yl)benylidene)amino]ethyl}pipera-zin-1-yl]amine] [84]. Each Pb(II) center on centrosymmetric dimers [Pb_2_(pbap)(NCS)_4_] and [Pb_2_(pbap)(N_3_)_4_] takes a partial square pyramidal shapes with ligating PbN_5_ chromophore with (N^p^, N^i^, N^a^) donor sets of pbap and two nitrogen atoms of the terminal pseudohalides (Figure 4).

Mirtamizdoust (2007) designed a simple manner for the preparation of two new 1D polymeric lead(II) coordination compounds containing the Pb_2_-(μ-N_3_)_2_ elements, namely [Pb(phen)(N_3_)_2_]_n_ (phen: 1,10-phenanthroline) and [Pb(deta)(N_3_).(N_3_)]_n_ (deta: diethylenetriamine) [85]. The coordination number in [Pb(phen)(N_3_)_2_]_n_ was six, and each lead atom was chelated by two nitrogen atoms of the phen ligand with Pb-N distances of 2.506(6) and 2.514(5) Å, and four nitrogen atoms of the azide anions with Pb-N distances of 2.418(6), 2.611(6), 2.706(5), and 2.816(5) Å. The coordination number in [Pb(deta)(N_3_).(N_3_)]_n_ was five, and each lead atom was chelated by three nitrogen atoms of the “deta” ligand with Pb-N distances of 2.457(6), 2.465(6), and 2.65(6) Å, and two nitrogen atoms of the azide anions with Pb-N distances of 2.905(8) and 2.905(8) Å. They were both assumed to have a hemidirected coordination geometry. Furthermore, these 1D CPs were the new Pb_2_-(μ-N_3_)_2_ elements of Pb(II) complexes and interreacted with each other through pi…pi stacking and weak Pb^_ _ _^N relations for creating 2D and 3D structures.

**Figure 5:**
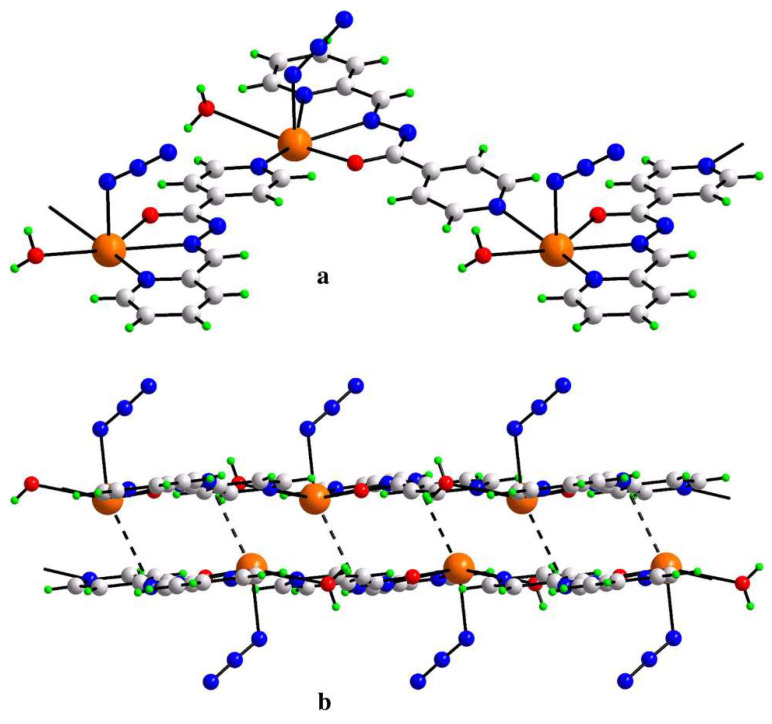
(**a**) A piece of the CP indicating the one-dimensional (1D) zig-zag polymeric chain and (**b**) the relation between chains linked with dual symmetry through Pb–N3 relations. Reprinted with permission from [86]. Copyright 2015, Springer Nature.

Mirtamizdoust et al. (2016) reported a simple route for the preparation of Pb(II) CP with a terminal azide ion [Pb(µ-2-pinh)N_3_H_2_O]_n_ (2-pinh: 2-pyridinecarbaldehyde isonicotionoyhydrazone) using an ultrasonic method [86]. As shown in Figure 5, the complex crystal structure formed a 1D zig-zag polymer in solid state. The coordination number of Pb(II) ion was six (PbN_4_O_2_). This coordination contained one oxygen atom and three N atoms from two organic linker ligands, one oxygen atom from coordinated water, and one nitrogen atom from a terminal coordinated azide anion. The prepared [Pb(µ-2-pinh)N_3_H_2_O]_n_ was applied to the synthesis of PbO NPs by thermolysis of the nanocomplex with oleic acid (OA) as a surfactant at 180 °C.

**Figure 6:**
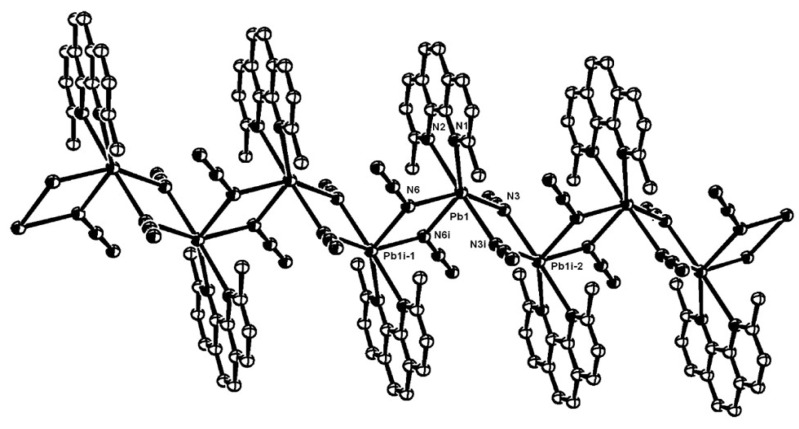
A piece of the CP indicating a 1D polymer. Reprinted with permission from [87]. Copyright 2009, WILEY-VCH Verlag GmbH & Co. KGaA, Weinheim.

In a study conducted by Shaabani’s group, a new 1D Pb(II) CP containing Pb_2_-(μ-N_3_)_2_ element [Pb(dmp)(N_3_)_2_]_n_ (dmp: 2,9-dimethyl-1,10-phenanthroline) was synthesized and identified [87]. According to single-crystal XRD, the coordination number of Pb(II) ions was six (PbN_6_), and it had the hemidirected coordination space (Figure 6). The lead atoms were linked by two nitrogen atoms of the “dmp” ligands with Pb-N distances of 2.699(2) and 2.673(1) Å, and four nitrogen atoms of the azide anions with Pb-N distances of 2.502(2), 2.529(2), 2.841(2), and 2.803(2) Å. Azide ions showed the coordination states as a μ_1,1_ end-to-on mode. They showed that the title complex’s HOMO was mainly restricted between two N atoms of one azide anion. Simultaneously, the LUMO was delocalized almost on all “dmp” ligand atoms having azide anions and Pb(II).

**Figure 7:**
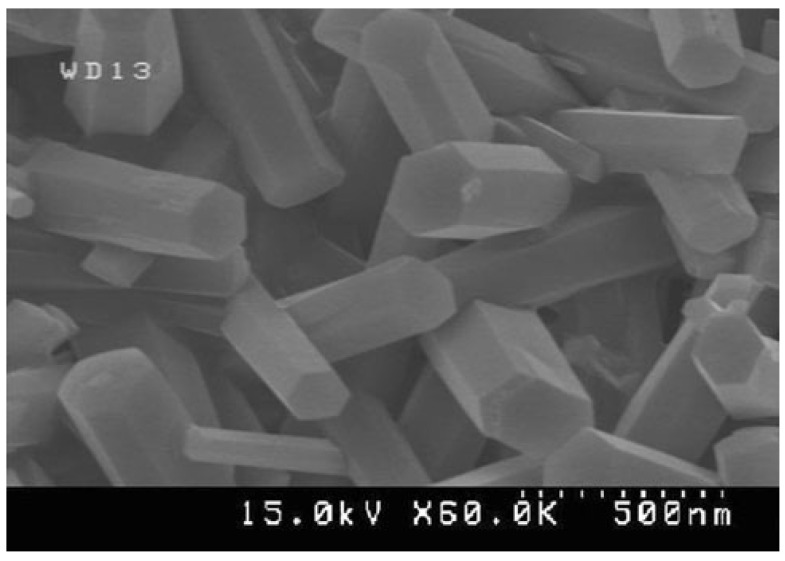
SEM images of the nano-hexagonal rods. Reprinted with permission from [88]. Copyright 2012, Springer Nature.

**Figure 8:**
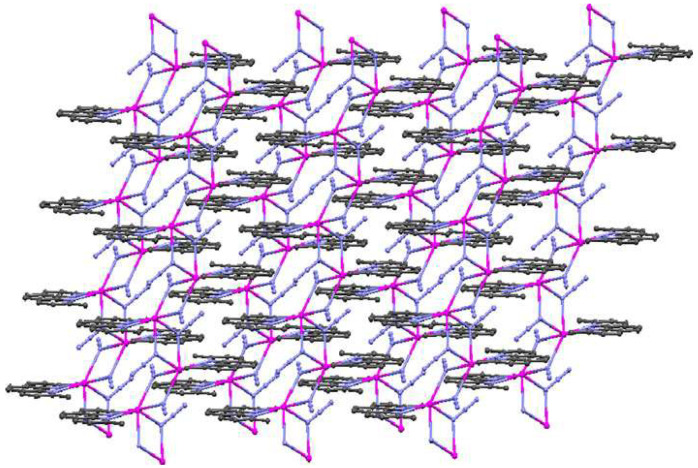
Packing of 1D chains for the formation of 3D supramolecular layers through pi…pi stacking relations. Reprinted with permission from [88]. Copyright 2012, Springer Nature.

Joo et al. (2012) developed a simple manner for the preparation of micro-hexagonal bars of a novel 1D polymeric Pb(II) coordination polymer containing Pb_2_-(μ-N_3_)_2_ pattern ([Pb(dmp)(N_3_)_2_]_n_) (dmp: 2,9-dimethyl-1,10-phenanthroline) by the sonochemical method (Figure 7) [88]. According to Figure 7, Species 1 contains rods with a diameter of 90 nm. There is a need for a further investigation of how this structure forms and whether or not it is influenced by the crystal structure of the compound, which has a one-dimensional rod-like structure. A heat gradient was imposed on a reagent solution, and it was used to obtain a single crystalline material. As demonstrated by the XRD results, the coordination number of lead(II) ions was six (PbN6) and had stereochemically active electron lone pairs. These results also showed a hemidirected coordination space. The azide ions’ coordination states were μ_1,1_ end-to-on. Moreover, it was observed that the chains had interactions via pi…pi stacking relations, which led to the creation of a 3D structure (Figure 8).

**Figure 9:**
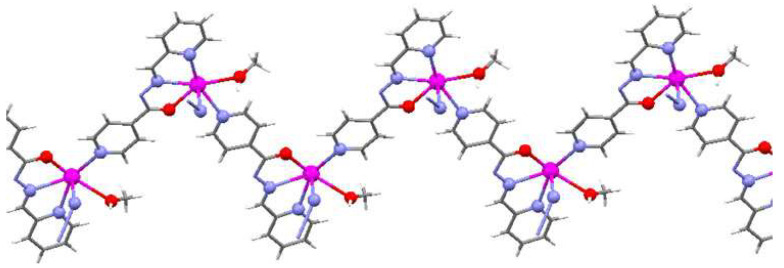
Piece of CP indicating a 1D zig-zag polymer. Reprinted with permission from [81]. Copyright 2013, Springer Nature.

**Figure 10:**
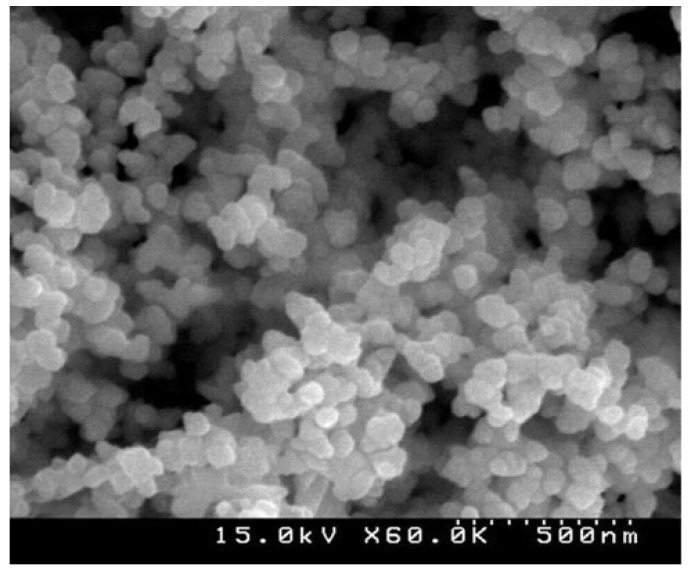
SEM images of PbO nanopowders created by [Pb(pcih)N_3_MeOH]_n_ calculations. Reprinted with permission from [89]. Copyright 2013, Springer Nature.

Shin and Min (2013) reported a straightforward method for the preparation of the first nanostructure lead(II) coordination complex, [Pb(pcih)N_3_MeOH]_n_ (pcih: 2-pyridinecarbaldehyde) with a sonochemical process [89]. Single-crystal X-ray crystallography was used to determine the geometry of this structure. According to the obtained results, the compound had a shape of a zig-zag 1D polymer with a Pb(II) ions’ coordination number of six, (PbN_4_O_2_) in the solid-state, with one O donor, three N donor, atoms from one O donors, two “pcih” from MeOH, and one nitrogen atom from the terminal azide anion (Figure 9). Then, thermolysis of CP by OA at 180 °C led to the production of a pure-phase nanosized PbO. SEM was used to determine the size and morphology of the synthesized Pb(II) oxide samples (Figure 10). As shown in Figure 10, [Pb(pcih)N_3_MeOH]_n_ was calcined to produce PbO nanopowders. The bulk powder of [Pb(pcih)N_3_MeOH]_n_ produced regularly shaped Pb(II) oxide nanoparticles with a diameter of about 35 nm.

**Figure 11:**
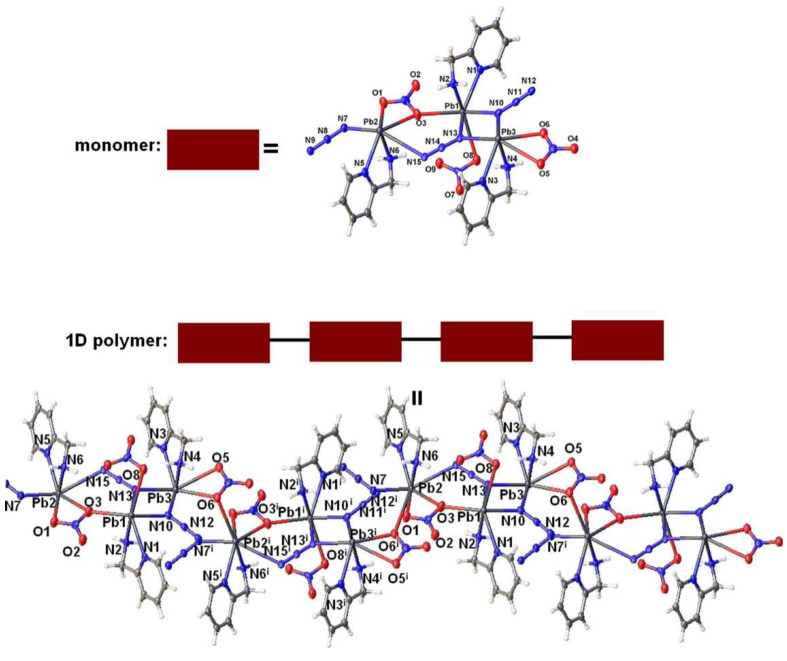
From 3 nuclear building blocks (monomer) to 1D CP. Reprinted with permission from [90]. Copyright 2015, Springer Nature.

Shahverdizadeh (2015) reported an efficient approach for the synthesis of a novel Pb(II) CP [Pb_3_(p-2ma)_3_(N_3_)_3_(NO_3_)_3_]_n_ (p-2ma: pyridine-2-ylmethanamine) using the sonochemical process [90]. The coordination complex was a polymer in the solid-state in the form of a 1D coordination (Figure 11). Three lead(II) centers were present by various coordination numbers. Moreover, three attractive coordination states of azide anions (μ_2_-1,1, μ_3_-1,1,3, and terminal azide anions) were found in the configuration. Each Pb1 atom was coordinated by two nitrogen atoms of two “p-2ma” ligands with Pb-N distances of 2.660(18) and 2.450(20) Å, one oxygen atom of a nitrate-bridged anion with a Pb-O distance of 2.699(14), one oxygen atom of the terminal nitrate anion with a Pb-N distance of 2.997(6) and two nitrogen atoms of two azide bridged anions with Pb-N distances of 2.415(19) and 2.516(18) Å, with a PbN_4_O_2_ donor set. Each Pb2 atom was coordinated by two nitrogen atoms of two “p-2ma” ligands with Pb-N distances of 2.585(19) and 2.460(20) Å, three oxygen atoms of nitrate-bridged anions with Pb-O distances of 2.732(14), 2.711(16), and 2.998(14), one nitrogen atom of the terminal azide anion with a Pb-N distance of 2.363(18) and one nitrogen atom of two azide-bridged anion with Pb-N distances of 2.942(18) Å, with a PbN_4_O_3_ donor set. Each Pb3 atom was coordinated by two nitrogen atoms of two “p-2ma” ligands with Pb-N distances of 2.626(18) and 2.430(20) Å, two oxygen atoms of nitrate-bridged anion with Pb-N distances of 2.711(14) and 2.908(12), two nitrogen atoms of two azide-bridged anions with Pb-N distances of 2.496(19) and 2.559(16) Å, with a PbN_4_O_2_ donor set. The synthesized [Pb_3_(p-2ma)_3_(N_3_)_3_(NO_3_)_3_]_n_ was used in the preparation of PbO NPs by thermolysis of a nanocomplex with OA as a surfactant at 180 °C.

**Figure 12:**
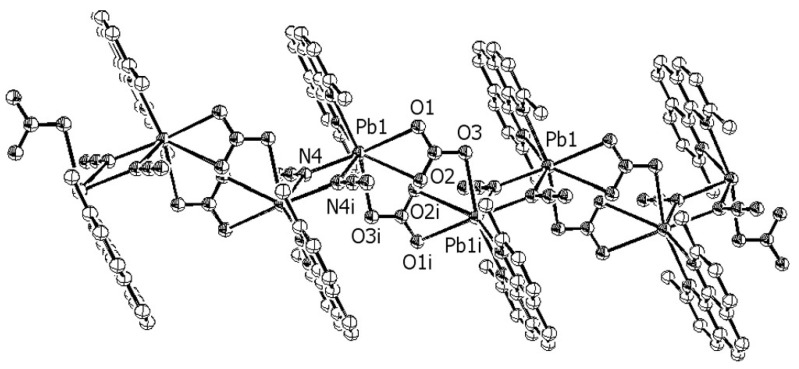
Piece of the CP indicating the 1D polymer. Reprinted with permission from [91]. Copyright 2011, WILEY-VCH Verlag GmbH & Co. KGaA, Weinheim.

Shaabani et al. (2011) proposed a straightforward manner for the synthesis of the Pb(II) coordination compound [Pb(dmp)(μ-N_3_)(μ-NO_3_)]*_n_* (dmp: 2,9-dimethyl-1,10-phenanthroline) utilizing a sonochemical procedure [91]. The single-crystal analysis showed that the coordination number of lead(II) was seven (PbN_4_O_3_), having an electron lone pair with stereochemical activity, and the coordination sphere was hemidirected. Each lead atom was chelated by two nitrogen atoms of “dmp” with Pb-N distances of 2.647(3) and 2.636(4) Å, two azide anions with Pb-N distances of 2.413(4) and 2.554(4) Å, and three nitrate oxygen atoms with Pb-O distances of 2.975(5), 2.761(4), and 2.761(4) Å (Figure 12). Single-crystal X-ray analysis showed that the complex crystallized in the triclinic system with space group *P*1, taking the form of a one-dimensional polymer in the solid-state. The Scherrer formula was used for estimating the particle average size, which was obtained as 65 nm.

**Figure 13:**
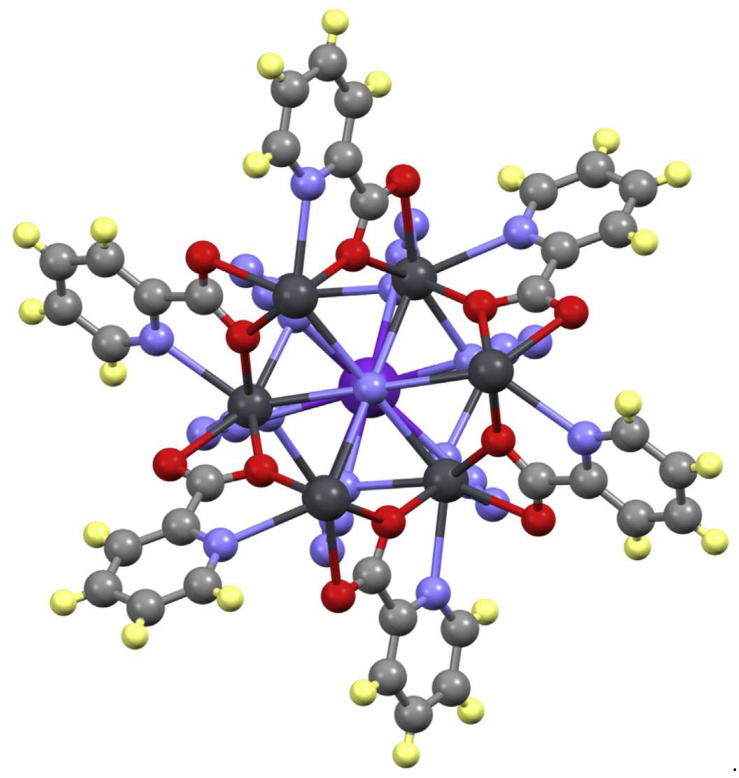
C axis indicates the lead atoms’ coordination. Reprinted with permission from [92]. Copyright 2010, Elsevier Ltd., Amsterdam, The Netherlands.

Gutierrez et al. (2017) developed a facile procedure for the preparation of NPs of one novel Pb and K coordination compound {[Pb_6_(pyc)_6_(N_3_)_7_K].½H_2_O}_n_ (pyc: 2-picolinate, C_6_H_4_NO_2_^−^) using the sonochemical method [92]. The polymer’s crystal structure consisted of a 1D CP complex, in which the lead ion had a coordination number of seven (Figure 13). All the picolinate groups bridged two adjacent lead atoms in a double chelating way. The picolinate was bonded through the pyridine nitrogen and one carboxylate oxygen atom to the first lead, while also chelating the second lead atom through the bidentate carboxylate group. The bond distances between 2.41 and 2.78 Å were in the range typical for Pb-O and Pb-N bonds and the angles between Pb-O-Pb and Pb-N-Pb were 145.88° and 109.94°, respectively.

**Figure 14:**
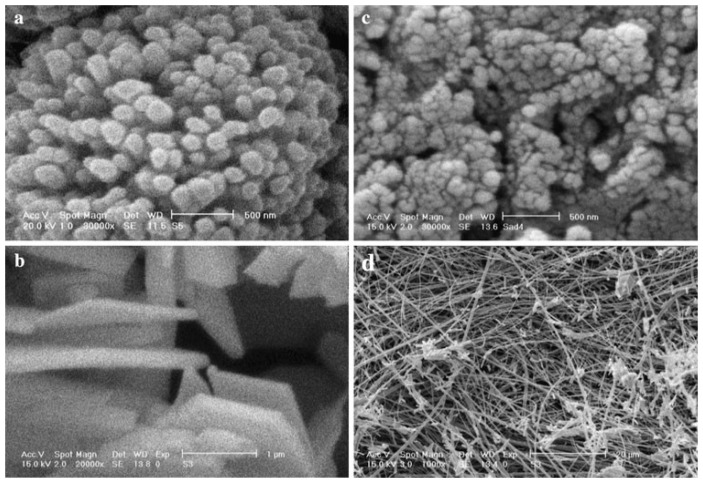
SEM images of (**a**) [Pb(3-pyc)(N_3_)(H_2_O)]_n_ (1) nanostructure balls in aqua solution, (**b**) [Pb(3-pyc)(N_3_)(H_2_O)]_n_ (1) nanoplates inethanolic solution, (**c**) [Pb(3-pyc)I]_n_ (2) NPs, and (**d**) [Pb(3-pyc)Br]_n_ (3) nanofibers. Reprinted with permission from [93]. Copyright 2010, Elsevier Ltd., Amsterdam, The Netherlands.

Sadeghzadeh and Morsali (2010) reported a facile and straightforward approach for the synthesis of nanostructures of three novel Pb(II) CPs, [Pb(3-pyc)I]_n_, [Pb(3-pyc)Br]_n_, and [Pb(3-pyc)(N_3_)(H_2_O)]_n_, (3-pyc: 3-pyridine carboxylic acid) with a sonochemical process (Figure 14) [93]. [Pb(3-pyc)I]_n_ was composed of rods with sizes of about 60 nm, with all rods arranged to form a hedge ball network (Figure 14a). The morphologies of compounds [Pb(3-pyc)Br]_n_ and [Pb(3-pyc)(N_3_)(H_2_O)]_n_ were nanoparticles and nanofibers, respectively (Figure 14c,d). Differences between these morphologies may be because of different packing structures and their diverse ligands. Although a further study is needed into how these network structures formed, they were likely caused by the crystal structure of the compounds, which may have influenced the morphology of the nanostructures of compounds 1–3. The single-crystal XRD results revealed compounds [Pb(3-pyc)I]_n_ and [Pb(3-pyc)Br]_n_, which contained 2D and 3D CPs, respectively. However, the analysis did not provide an appropriate single crystal in the complex [Pb(3-pyc)(N_3_)(H_2_O)]_n_. The crystal structures of these coordination complexes showed that the coordination number of lead (II) ions in compounds [Pb(3-pyc)I]_n_ and[Pb(3-pyc)Br]_n_ was seven. This route’s significant advantages were the need for shorter reaction times and CP production by better outcomes and nano sizes.

**Figure 15:**
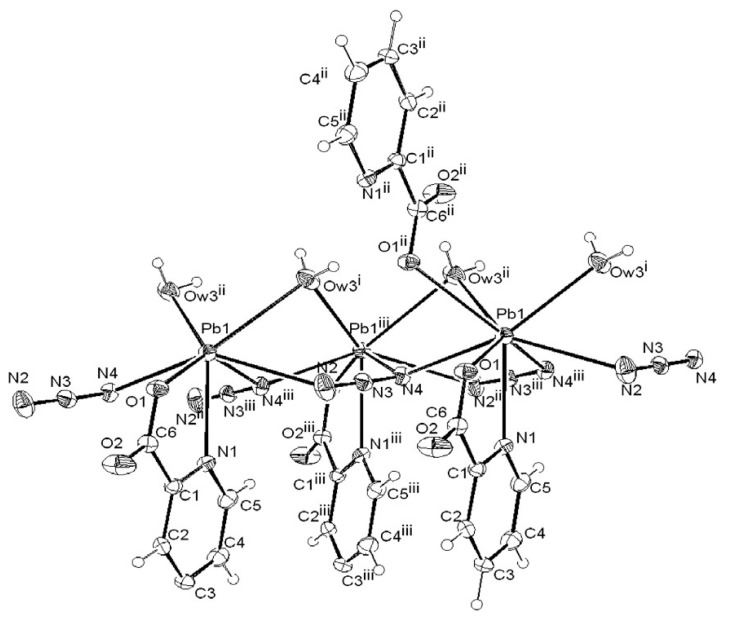
ORTEP image of complex [Pb(2-pyc)(N_3_)(H_2_O)]_n_. i: −x, y + 1/2, −z + 1/2; ii: −x, −y, −z; iii: −x, −y + 1/2, z + 1/2. Reprinted with permission from [81]. Copyright 2010, Springer Nature.

**Scheme 6:**
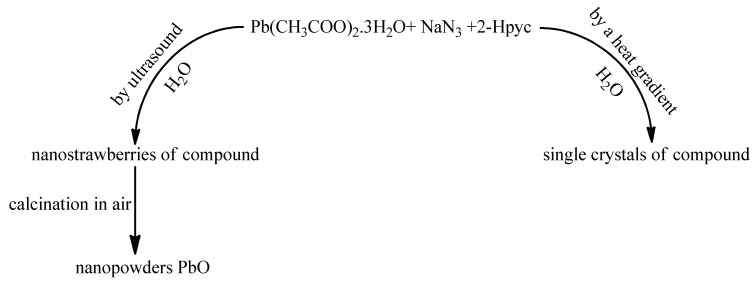
Materials produced and synthetic manners.

Morsali et al. (2010) reported a straightforward manner for the preparation of the nanostructure of a novel Pb(II) 2D coordination compound [Pb(2-pyc)(N_3_)(H_2_O)]_n_ (2-pyc: 2-pyridine carboxylic acid) with sonochemical irradiation [81]. The [Pb(2-pyc)(N_3_)(H_2_O)]_n_ crystal structure had a 2D polymer and showed that Pb(II) ions had a coordination number of seven. In the [Pb(2-pyc)(N_3_)(H_2_O)]_n_ compound, the building blocks of [Pb_2_(µ-H_2_O)_2_] were bridged by the azide anion and 2-pyc^−^ ligands, and each lead atom was coordinated by three oxygen atoms of the 2-pyc^−^ ligands, two oxygen atoms of the water molecules and three nitrogen atoms of the azide anions, as demonstrated in Figure 15. A PbO nanopowder was obtained by calcinating the nanostructure of CP at 400 °C (Scheme 6).

**Figure 16:**
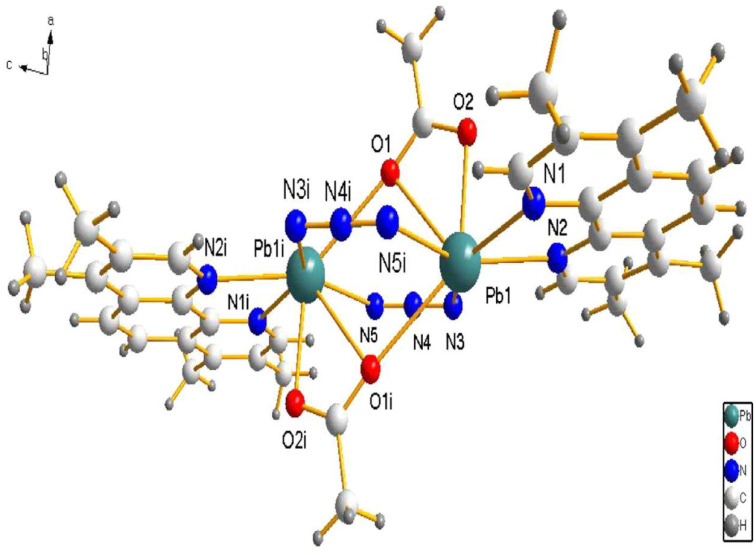
Molecular configuration of [Pb_2_(tmph)_2_(μ-N_3_)_2_(CH_3_COO)_2_]. Reprinted with permission from [94]. Copyright 2015, Elsevier Ltd., Amsterdam, The Netherlands.

**Figure 17:**
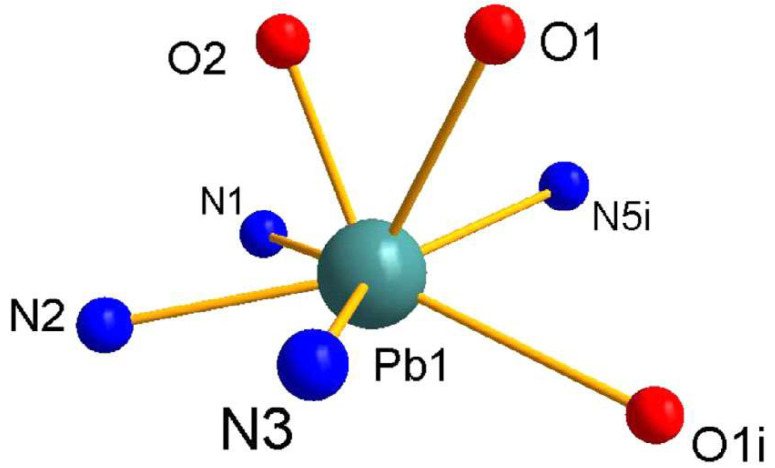
Schematic view of Pb(II) space. Reprinted with permission from [94]. Copyright 2015, Elsevier Ltd., Amsterdam, The Netherlands.

Mirtamizdoust (2014) designed a simple procedure for the synthesis of the novel natural binuclear Pb(II) azido CP ([Pb_2_(tmph)_2_(μ-N_3_)_2_(CH_3_COO)_2_]_n_) (tmph: 3,4,7,8-tetramethyl-1,10-phenanthroline) by sonochemical irradiation [94]. The coordination complex had a bridging azido path and end-to-end linking azides among a pair of Pb(II) centers (Figure 16). The coordination number of Pb(II) ions was seven (PbN_4_O_3_), by three O donors from the acetate anions, two N donor atoms from the tmph ligands, and two N-donors from two azide anions (Figure 17). Thermolyzing **1** with OA as a surfactant at 180 °C resulted in obtaining the Pb(II) oxide NPs.

**Figure 18:**
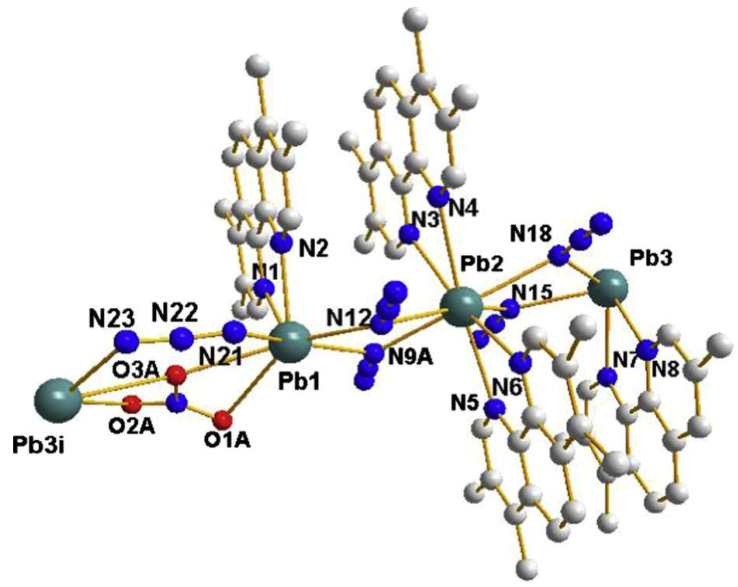
[Pb_3_(tmph)_4_(µ-N_3_)_5_(µ-NO_3_)] molecular structure (A = i = −x, −y, −z). Reprinted with permission from [95]. Copyright 2015, Elsevier Ltd., Amsterdam, The Netherlands.

Hanifehpour et al. (2015) reported a practical approach for the sonochemical synthesis of the rod-formed nanostructure of a novel 1D Pb(II) trinuclear coordination compound that contained Pb_2_-(µ-N_3_)(NO_3_) and Pb_2_-(µ-N_3_) motifs [Pb_3_(tmph)_4_(µ-N_3_)_5_(µ-NO_3_)]_n_ (tmph: 3,4,7,8-tetramethyl-1,10-phenanthroline) [95]. As shown by a single-crystal XRD analysis, three Pb(II) centers were observed by coordination numbers eight and seven with hemidirected and holodirected coordination geometries (Figure 18). As shown in Figure 18, there were three different lead(II) centers in the structure. The Pb1 atom was coordinated by two oxygen atoms of the bridged nitrate anions with the Pb-O distances of 2.845(2) and 2.894(2) Å, two nitrogen atoms of the µ_−1,1_ bridged azide anions with the Pb-N distances of 2.545(9) and 2.523(11) Å, one nitrogen atoms of the µ_-1,3_ bridged azide anion with the Pb-N distance of 2.764(12) and two nitrogen atoms of the “tmph” ligands with the Pb-N distances of 2.483(8) and 2.461(8) Å, in a seven fashion, with a PbN_5_O_2_ donor set. The Pb2 atom was coordinated by four nitrogen atoms of four µ_−1,1_ bridged azide anions with the Pb-N distances of 2.635(11), 2.682(10), 2.698(10), and 2.684(9) Å, and four nitrogen atoms of two “tmph” ligands with the Pb-N distances of 2.869(8), 2.702(8), 2.736(8), and 2.766(8) Å, in an eight fashion, with a PbN_8_ donor set. The Pb3 atom was coordinated by two oxygen atoms of the bridged nitrate anions with the Pb-O distances of 2.885(12) and 2.891(12) Å, two nitrogen atoms of two µ_−1,1_ bridged azide anions with the Pb-N distances of 2.601(10) and 2.500(10) Å, one nitrogen atoms of the µ_−1,3_ bridged azide anions with the Pb-N distance of 2.710(12), and two nitrogen atoms of the “tmph” ligands with the Pb-N distances of 2.474(8) and 2.533(8)Å, in a seven fashion, with a PbN_5_O_2_ donor set. Moreover, these authors illustrated that a 3D framework was created through pi…pi stacking the chains’ interactions. They used the developed CP to prepare PbO NPs by thermolysis of the nanocomplex with OA as a surfactant at 180 °C.

**Scheme 7:**
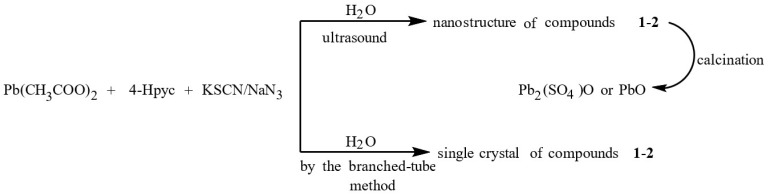
Produced materials and synthetic approaches.

A simple procedure was reported by Morsali and Soltanian (2010) to synthesize nanostructures of two novels Pb(II) 2D CPs [Pb(μ-4-pyc)(μ-NCS)-(μ-H_2_O)]_n_ and [Pb(μ-4-pyc)(μ-N_3_)(μ-H_2_O)]_n_ (4pyc: 4-pyridine carboxylic acid) based on a thermal gradient method using a sonochemical approach (Scheme 7) [80]. The structure of [Pb(μ-4-pyc)(μ-NCS)-(μ-H_2_O)] may also be considered as a coordination polymer of lead(II) consisting of units with a building block of [Pb_2_(µ-H_2_O)_2_] where the SCN^−^ and 4-pyc^−^ anions bridge two lead(II) ions via the N and O atoms. The coordination number in [Pb(μ-4-pyc)(μ-NCS)-(μ-H_2_O)]_n_ was seven, and each lead atom was coordinated by two oxygen atoms of the 4-pyc^−^ ligands with Pb-O distances of 2.467(3) and 2.711(3) Å, two oxygen atoms of the water molecules with Pb-O distances of 2.578(3) and 2.905(3) Å, and two nitrogen atoms of the thiocyanate anion with Pb-N distances of 2.695(4) and 2.850(4) Å. In [Pb(μ-4-pyc)(μ-N_3_)(μ-H_2_O)]_n_, the building block of [Pb_2_(µ-H_2_O)_2_] was bridged by the azide and 4-pyc^−^ anions. The coordination number in [Pb(μ-4-pyc)(μ-N_3_)(μ-H_2_O)]_n_ was eight, and each lead atom was coordinated by two oxygen atoms of the 4-pyc^−^ ligands with Pb-O distances of 2.518(3) and 2.672(3) Å, two oxygen atoms of the water molecules with Pb-O distances of 2.629(3) and 2.672(3) Å, and four nitrogen atoms of the azide anions with Pb-N distances of 2.751(4), 2.754(4), 2.779(4), and 3.029(5) Å. Single-crystal XRD was used for the characterization of complexes [Pb(μ-4-pyc)(μ-NCS)-(μ-H_2_O)]_n_ and [Pb(μ-4-pyc)(μ-N_3_)(μ-H_2_O)]_n_, which contained 2D polymeric elements. PbO and Pb_2_(SO_4_)O NPs were obtained by calcinating nanostructures of complexes [Pb(μ-4-pyc)(μ-NCS)-(μ-H_2_O)]_n_ and [Pb(μ-4-pyc)(μ-N_3_)(μ-H_2_O)]_n_ at 600 °C.

**Scheme 8:**
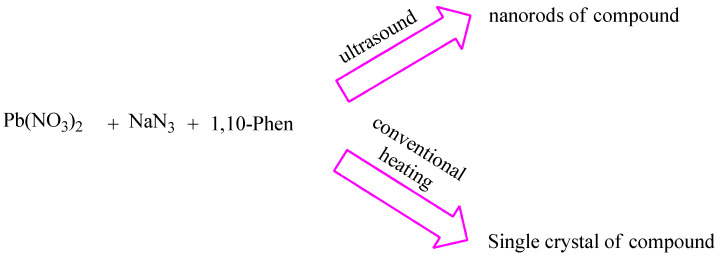
Produced materials and synthetic approaches.

**Figure 19:**
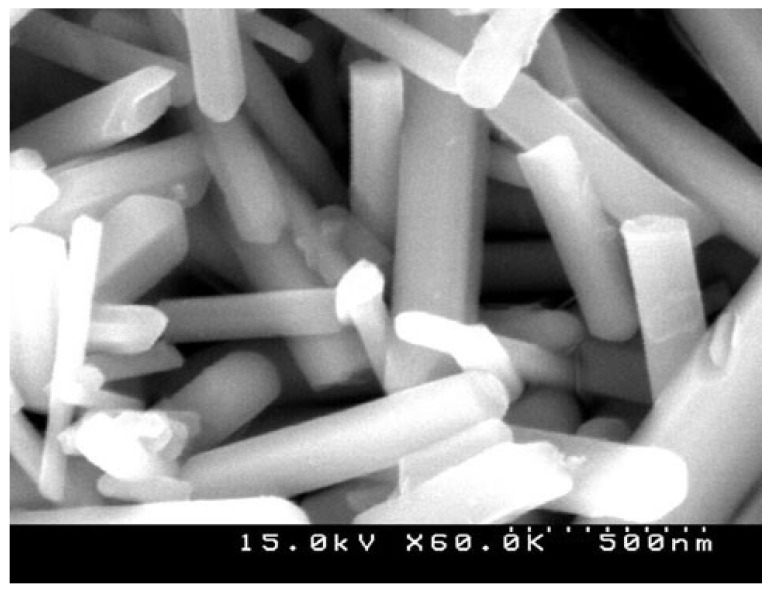
SEM photographs of [Pb(phen)(μ-N_3_)(μ-NO_3_)]_n_ nanorods obtained from ultrasonic radiation. Reprinted with permission from [96]. Copyright 2012, Springer Nature.

Hanifehpour (2010) offered a simple procedure for the synthesis of nanorods of a new 1D polymeric Pb(II) coordination complex with Pb_2_-(μ-N_3_)_2_ pattern ([Pb(phen)(μ-N_3_)(μ-NO_3_)]_n_) (phen: 1,10-phenanthroline) using a sonochemical manner [96]. The sonochemically prepared compound (Figure 19) had a rod-like morphology with a thickness of about 58 nm. The formation mechanism of this structure may be influenced by the compound’s one-dimensional rod-like crystal structure, which requires further study. A heat gradient was imposed on a reagent solution and used to obtain a single crystalline material (Scheme 8). According to the single-crystal XRD results, the coordination number of the lead(II) ions was eight (PbN_4_O_4_) by the Pb(II) ions containing “stereochemically active” electron lone pairs. Each lead atom was chelated by two nitrogen atoms of phen with Pb-N distances 2.53(1) and 2.50(1) Å; two azide anions with Pb-N distances of 2.44(1) and 2.74(1) Å; and four nitrate oxygen atoms with Pb-O distances of 2.75(1), 2.86(7), 2.87(9), and 2.98(1) Å. The structure showed a hemidirected coordination space. Thermolyzing ([Pb(phen)(μ-N_3_)(μ-NO_3_)]_n_ with OA as a surfactant at 180 °C yielded PbO NPs.

**Figure 20:**
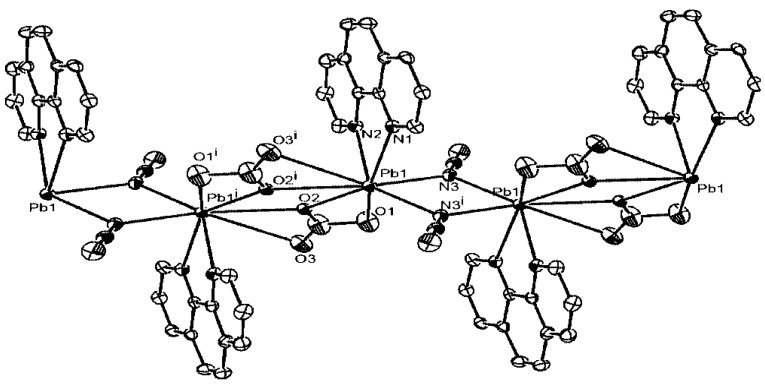
A piece of the CP indicating a 1D polymer. Reprinted with permission from [97]. Copyright 2006, WILEY-VCH Verlag GmbH & Co. KGaA, Weinheim.

Marandi et al. (2016) proposed a facile route for the synthesis of a new 1D polymeric Pb(II) compound having the Pb_2_-(μ-N_3_)_2_ pattern of [Pb(phen)(μ-N_3_)(μ-NO_3_)]_n_ (phen: 1,10-phenanthroline) [97]. As illustrated by the single crystal XRD results, the coordination number of lead(II) ions was eight (PbN_4_O_4_), and the Pb(II) ions contained “stereochemically active” electron lone pairs. The results also showed a hemidirected coordination space (Figure 20). Each lead atom was chelated by two nitrogen atoms of phen with Pb-N distances of 2.53(1), 2.5(1) Å, two azide anions with Pb-N distances of 2.44(1), 2.74(1) Å, and four nitrate oxygen atoms with Pb-O distances of 2.75(1), 2.86(7), 2.87(9), and 2.98(1) Å. Table 1 provides other reports for the synthesis of lead–azide coordination polymer.

## 4. Use of Lead Azide CPs as Precursors for PbO Preparation

Lead-azido CPs serve as appropriate precursors for preparing favorable materials at the nanoscale. Using these polymers as precursors is a beneficial approach for preparing lead oxides. Some advantages of this approach include simple processing, no need for specific tools, the relation between raw materials and the target products’ structures, higher suitability for products’ phase control; higher control of purity, particle size, process situations, and particle crystal; decreasing the possibility of interparticle collisions, straightforwardness, being economical, and the large-scale production potential [105]. Additionally, the capping ligand presence can avoid the unfavorable accumulation of nano products. Another advantage of this approach is the desired controllability of elemental components achieved by combining the suitable organic linking ligands and the designated metal ions. The ultimate morphology mechanism in nanomaterials probably depends on some intermediates controlled by external and internal forces within the formation procedure. MOCP interactions and crystal structure (e.g., covalence, hydrogen, van der Waals forces, and coordination) are internal forces that affect solvent-MOCP. Moreover, intermediates interactions, dipolar and electrostatic fields, and hydrophilic or hydrophobic interactions cause exterior forces to control the system’s morphology [106,107,108,109]. Subsequently, the size and morphology of inorganic nanomaterials depend on different factors, including the preparation approach [110,111], the relation between the morphology of favorite materials and MOCP crystalline structure [112,113,114,115,116], surfactant impact [117,118,119,120], the temperature of thermolysis [121,122,123,124,125], size of initial MOCP precursors [126,127], and impact of the MOCP precursors’ initial morphology [128,129,130,131,132,133,134]. The major challenge is the precise and explicit mechanisms for the MOCPs transformation to needed nanomaterials. Future studies should focus on describing and explaining nano-materials formation mechanisms and discovering rules governing the relations among affecting factors and ultimate morphologies. It can be realized using different MOCPs with varying structural varieties and compositions (e.g., 1D, 2D, and 3D compositions) and porous and nonporous constructions. Table 2 indicates various examples of lead azide polymer compounds from which lead oxide is developed.

**Figure 21:**
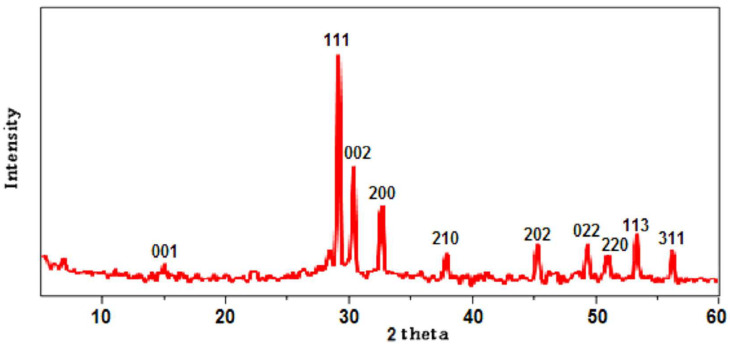
XRD pattern of PbO prepared after thermolysis of compound [Pb2(tmph)2(µ-N3)2(CH3COO)2]. Reprinted with permission from [94]. Copyright 2015, Elsevier Ltd., Amsterdam, The Netherlands.

**Figure 22:**
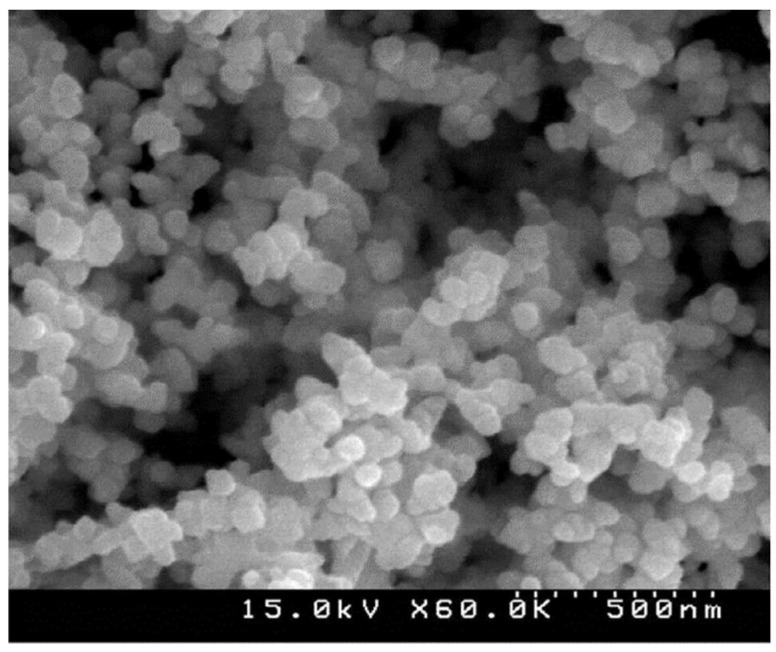
SEM photographs of PbO nanopowders (produced by thermolysis of nanorods). Reprinted with permission from [94]. Copyright 2015, Elsevier Ltd., Amsterdam, The Netherlands.

**Figure 23:**
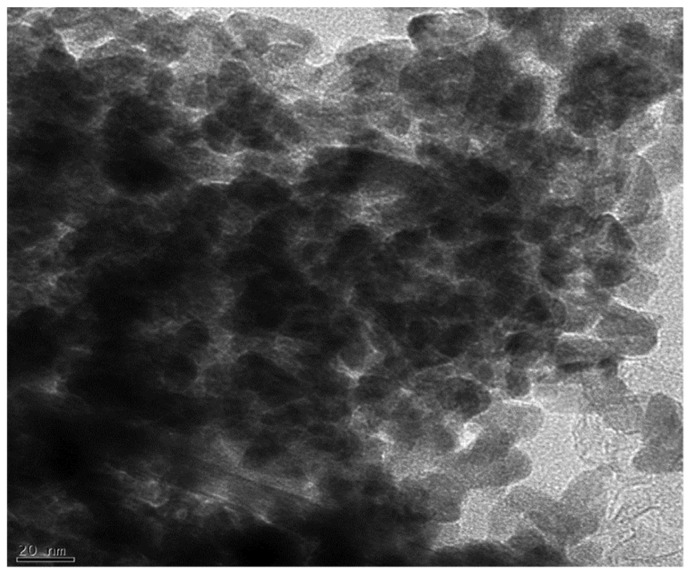
TEM photographs of PbO nanopowders (produced by thermolysis of nanorods). Reprinted with permission from [95]. Copyright 2015, Elsevier Ltd., Amsterdam, The Netherlands.

**Figure 24:**
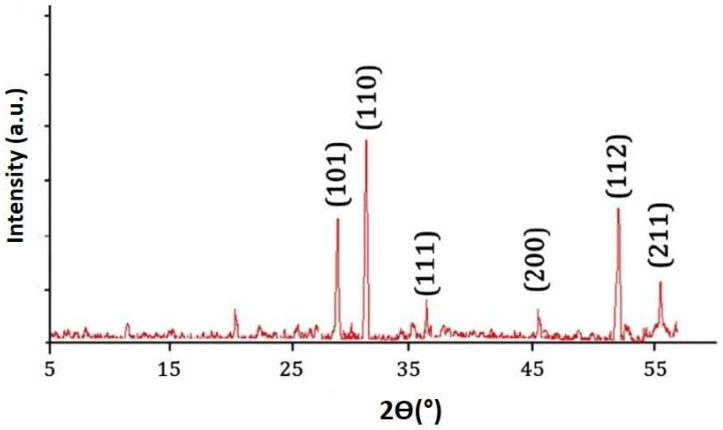
XRD pattern of PbO prepared after thermolysis of compound [Pb3(tmph)4(µ-N3)5(µ-NO3)]n. Reprinted with permission from [95]. Copyright 2015, Elsevier Ltd., Amsterdam, The Netherlands.

As shown in Section 3, the nanopowders of PbO were obtained from the decomposition of the precursor [Pb_2_(tmph)_2_(µ-N_3_)_2_(CH_3_COO)_2_]_n_ in oleic acid as a surfactant at 180 °C, under an air atmosphere [93]. Figure 21 shows the XRD pattern of orthorhombic PbO with α = 5.8931 Å and z = 4. The morphology and size of the as-prepared PbO samples were further investigated using scanning electron microscopy (SEM). This process produced a regular shape of lead(II) oxide nanoparticles with a diameter of about 35–50 nm (Figure 22). Moreover, the nanopowders of PbO synthesized by decomposition of the precursor [Pb_3_(tmph)_4_(µ-N_3_)_5_(µ-NO_3_)]_n_ in oleic acid as a surfactant at 180 °C, under an air atmosphere [94]. The morphology and size of the as-prepared PbO samples were investigated using a transmission electron microscope (TEM). This process produced a regular shape of lead(II) oxide nanoparticles with a diameter of about 10–20 nm (Figure 23). The final products obtained by decomposing the compound [Pb3(tmph)4(µ-N3)5(µ-NO3)]n, based on their XRD patterns (Figure 24), match with the standard pattern of tetragonal PbO with α = 3.947 Å, c = 4.988 Å, and Z = 2.

## 5. Conclusions

The purpose of the current study was to explain recent developments in the synthesis of Pb(II)-azido metal–organic coordination polymers. The first significant finding was that the coordination polymers have many applications and potential properties in many research fields, primarily dependent on particular host–guest interactions. The second finding was that the azido-bridged complexes are inorganic coordination ligands with higher fascination, which have been the subject of intense research because of their coordination adaptability and magnetic diversity. The other conclusions drawn from the present study are that Pb (II) has a wide variety of coordination numbers existing in its complexes because of its relatively large ion. One of the principal findings from this study was that in holodirected structures, the ligand atoms bond was scattered throughout the Pb(II) coordination space. However, in a hemidirected structure, the ligand atoms bond was diffused only in one part of the Pb(II) coordination space. The most obvious conclusion from this study is that CPs serve as appropriate precursors for preparing favorable materials at the nanoscale. Using these polymers as precursors is a beneficial approach for preparing inorganic nanomaterials such as metal oxides.

## Figures and Tables

**Table 1:** Other reports for the synthesis of lead–azide coordination polymer.

Molecular Formula	Coordination Number of Pb	Geometry of Pb	Modes Of Azide Ion	Structure of Polymer	Ref
[Pb(INO)(N_3_)(H_2_O)]_n_	seven	hemidirected	End to end	3D	[98]
[Pb_2_(phen)_2_(N_3_)_3_(ClO_4_)]_n_	eight	hemidirected	bridging	2D	[99]
[Pb(3-pyc)(N_3_)H_2_O]_n_	seven	-	bridging	2D	[100]
[Pb_2_(N_3_)_3_(NO_2_)(C_12_H_8_-N_2_)_2_]_n_	-	-	bridging	-	[101]
[Pb(PNO)(N_3_)]_n_	eight	hemidirected	End to end	2D	[102]
[Pb_2_(C_2_H_3_O_2_)_2_(N_3_)_2_(C_18_H_12_N_6_)_2_]	seven	hemidirected	bridging	-	[103]
[Pb(C_6_H_4_NO_2_)(N_3_)(H_2_O)]_n_	seven	-	bridging	2D	[104]

**Table 2:** Various examples of lead–azide polymers from which PbO is synthesized.

Molecular Formula	Coordination Number of Pb	Geometry of Pb	Modes of Azide Ion	Structure of Polymer	Ref
[Pb_2_(μ-N_3_)(μ-NO_3_)L_2_]_n_	Five	Hemidirected	Bridging	2D	[79]
[Pb(μ-2-pinh)N_3_H_2_O]_n_	Six	Hemidirected	End to end	1D	[86]
[Pb(pcih)N_3_MeOH]_n_	Six	Hemidirected	Bridging	1D	[89]
[Pb_3_(p-2ma)_3_(N_3_)_3_(NO_3_)_3_]_n_	Six	Hemidirected	Bridging	1D	[90]
[Pb(3-pyc)(N_3_)(H_2_O)]_n_	Seven	-	End to end	3D	[100]
[Pb(2-pyc)(N_3_)(H_2_O)]_n_	Seven	Holodirected	End to end	2D	[93]
[Pb_2_(tmph)_2_(μ-N_3_)_2_(CH_3_COO)_2_]_n_	Seven	Hemidirected	Bridging	1D	[95]
[Pb_3_(tmph)_4_(μ-N_3_)5(μ-NO_3_)]_n_	Eight	Hemidirected	Bridging	1D	[96]
[Pb(μ-4-pyc)(μ-N_3_)(μ-H_2_O)]_n_	Eight	Hemidirected	Bridging	2D	[80]
[Pb(phen)(μ-N_3_)(μ-NO_3_)]_n_	Eight	Hemidirected	Bridging	1D	[97]

## Data Availability

Not applicable.
